# Mg^2+^ block properties of triheteromeric GluN1–GluN2B–GluN2D NMDA receptors on neonatal rat substantia nigra pars compacta dopaminergic neurones

**DOI:** 10.1113/jphysiol.2013.267864

**Published:** 2014-05-15

**Authors:** Zhuo Huang, Alasdair J Gibb

**Affiliations:** 1 Department of Molecular and Cellular Pharmacology, State Key Laboratory of Nature and Biomimetic Drugs, Peking University School of Pharmaceutical SciencesBeijing, 100191, P.R. China; 2 Department of Neuroscience, Physiology and Pharmacology, University College LondonGower Street, London, WC1E 6BT, UK

## Abstract

Native NMDA receptors (NMDARs) are tetrameric channels formed by two GluN1 and two GluN2 subunits. So far, seven NMDARs subunits have been identified and they can form diheteromeric or triheteromeric NMDARs (more than one type of GluN2 subunit). Extracellular Mg^2+^ is an important regulator of NMDARs, and particularly the voltage dependence of Mg^2+^ block is crucial to the roles of NMDARs in synaptic plasticity and the integration of synaptic activity with neuronal activity. Although the Mg^2+^ block properties of diheteromeric NMDARs are fully investigated, properties of triheteromeric NMDARs are still not clear. Our previous data suggested that dopaminergic neurones expressed triheteromeric GluN1–GluN2B–GluN2D NMDARs. Here, using NMDARs in dopaminergic neurones from postnatal day 7 (P7) rats as a model system, we characterize the voltage-dependent Mg^2+^ block properties of triheteromeric NMDARs. In control conditions, external Mg^2+^ significantly inhibits the whole cell NMDA-evoked current in a voltage-dependent manner with IC_50_ values of 20.9 μm, 53.3 μm and 173 μm at −90 mV, −70 mV and −50 mV, respectively. When the GluN2B-selective antagonist ifenprodil was applied, the Mg^2+^ sensitivity of the residual NMDA-mediated currents (which is mainly carried by GluN1–GluN2B–GluN2D NMDARs) is reduced to IC_50_ values of 45.9 μm (−90 mV), 104 μm (−70 mV) and 276 μm (−50 mV), suggesting that triheteromeric GluN1–GluN2B–GluN2D NMDARs have less affinity for external Mg^2+^ than GluN1–GluN2B receptors. In addition, fitting *I*_NMDA_–*V* curves with a trapping Mg^2+^ block model shows the triheteromeric GluN1–GluN2B–GluN2D NMDARs have weaker voltage-dependent Mg^2+^ block (δ = 0.56) than GluN1–GluN2B NMDARs. Finally, our concentration jump and single channel recordings suggest that GluN1–GluN2B–GluN2D rather than GluN1–GluN2D NMDARs are present. These data provide information relevant to Mg^2+^ block characteristics of triheteromeric NMDARs and may help to better understand synaptic plasticity, which is dependent on these triheteromeric NMDARs.

## Introduction

NMDA receptors (NMDARs) are tetrameric, glutamate-gated monovalent cation and Ca^2+^-permeable channels that are expressed by nearly all mammalian neurones (Traynelis *et al*. 2010). To date, seven NMDAR subunits have been identified, i.e. GluN1, a group of GluN2 (GluN2A-GluN2D) and a pair of GluN3 subunits (GluN3A and GluN3B). Most native NMDARs appear to function as heteromeric assemblies composed of two GluN1 subunits and two GluN2 subunits and they form diheteromeric or triheteromeric NMDARs (which are formed with more than one type of GluN2 subunit) (Sheng *et al*. 1994; Dunah *et al*. 1998; Paoletti & Neyton, 2007; Traynelis *et al*. 2010).

NMDARs show a voltage-dependent Mg^2+^ block where extracellular Mg^2+^ inhibits NMDARs at negative membrane potentials and this blockage is relieved when the neurone is depolarized allowing NMDA receptors to function as coincidence detectors in many types of activity-dependent synaptic plasticity. Although it has been shown that diheteromeric NMDARs assembled from GluN1–GluN2A or GluN1–GluN2B subunits are blocked by extracellular Mg^2+^ more strongly than the channels formed by GluN1–GluN2C or GluN1–GluN2D subunits (Monyer *et al*. 1994; Kuner & Schoepfer, 1996; Qian *et al*. 2005; Retchless *et al*. 2012), the Mg^2+^ block properties of triheteromeric NMDARs are still not known.

In previous studies, we have reported that NMDARs on dopaminergic neurones of substantia nigra pars compacta (SNc) are composed of diheteromeric GluN1–GluN2B and triheteromeric GluN1–GluN2B–GluN2D NMDARs (Jones & Gibb, 2005; Brothwell *et al*. 2008; Suarez *et al*. 2010). Consistent with this, both GluN2B and GluN2D mRNA and protein are found in early development while no evidence for functional NR2A-containing NMDARs or NR2C protein or mRNA were found at these early postnatal ages (Monyer *et al*. 1994; Dunah *et al*. 1996, 1998). As the GluN2B-selective antagonist ifenprodil is more effective at blocking GluN1–GluN2B than GluN1– GluN2B-GluN2A NMDARs (Hatton & Paoletti, 2005), suggesting SNc neurones provide us with an ideal model system to investigate the voltage-dependent Mg^2+^ block of triheteromeric GluN1–GluN2B–GluN2D NMDARs.

In this study, using whole cell and single channel patch clamp electrophysiological recordings, we assessed the extracellular Mg^2+^ block properties of triheteromeric GluN1–GluN2B–GluN2D NMDARs in postnatal day 7 (P7) SNc dopaminergic neurones. We found that the degree of Mg^2+^ block was reduced during bath application of the GluN2B selective antagonist ifenprodil, showing that triheteromeric GluN1–GluN2B–GluN2D NMDARs display a weaker voltage-dependent Mg^2+^ block than diheteromeric GluN1–GluN2B NMDARs. In addition, quantitative simulations were used to infer the voltage dependence of Mg^2+^ block of these receptors. Our data show the unique Mg^2+^ block characteristic of triheteromeric GluN1–GluN2B–GluN2D NMDARs and may provide useful information relevant to NMDAR-dependent synaptic plasticity and help to constrain the parameters used in simulation studies.

## Methods

### Ethical approval

All animal protocols were performed in accordance with the Animals Scientific Procedures Act, UK (1986) and with UCL ethical approval.

Drugs were purchased from either (Sigma-Aldrich, Dorset, UK) (Abcam, Cambridge, UK) or (Fisher Scientific, Loughborough, UK) (BDH, Merck Ltd., Poole, UK). Aqueous stock solutions of NMDA (100 mm), glycine (10 mm), bicuculline methiodide (10 mm), tetrodotoxin (100 μm), strychnine (1 mm), DNQX (20 mm), nimodipine (2 mm) and conotoxin MVIIC (0.5 mm) were kept at −20°C until use.

### Slice preparation

Coronal brain slices (250–300 μm) were prepared from 6- to 8-day old (‘P7’) Sprague–Dawley rats in the manner described previously (Jones & Gibb, 2005; Suarez *et al*. 2010). Briefly, a rat was decapitated and the brain was quickly removed into ice-cold ‘slicing solution’ containing (mm): sucrose 206; KCl 2.5; CaCl_2_ 1.0; MgCl_2_ 1.0; NaHCO_3_ 25; NaH_2_PO_4_ 1; glucose 25 (bubbled with 95% O_2_ and 5% CO_2_, pH 7.4). Brain slices were prepared using a vibratome (Dosaka DTK-1000, Kyyoto, Japan) and kept in a holding chamber containing external recording solution of the following composition (mm): NaCl 125; KCl 2.5; CaCl_2_ 1.0; MgCl_2_ 4.0; NaHCO_3_ 26; NaH_2_PO_4_ 1.25; glucose 25 (bubbled with 95% O_2_ and 5% CO_2_, pH 7.4); at room temperature for 1–6 h before use.

### Identification of postnatal day 7 dopaminergic neurones

Dopaminergic neurones were identified using a combination of locational, morphological and electrophysiological criteria. Initially, dopaminergic neurones were distinguished from interneurones by their large cell bodies, which were ovoid, polygonal or fusiform in shape and emitted two to six primary dendrites from cell bodies (Fig. 1*C*). In a cell-attached gigaseal (>10 GΩ) configuration, dopaminergic neurones very often displayed spontaneous activity at a constant rate of 0.5–5.0 Hz (Tepper *et al*. 1987; Yung *et al*. 1991). In addition, in whole cell voltage clamp configuration, hyperpolarization-activated cyclic nucleotide-gated currents (*I*_h_ current) activated by voltage steps from −60 mV to −120 mV for 1.5 s were used to identify dopamine cells. The activation time course of these *I*_h_ currents was obtained by fitting the *I*_h_ current trace with a single exponential equation: *I*(*t*) = *A* exp(−*t*/τ) + *S*_s_ where *I*(*t*) is the current amplitude at any given time (*t*), *A* is the peak amplitude of the *I*_h_ current, τ is the decay time constant and *S*_s_ the amplitude of steady-state *I*_h_ current. Neurones with amplitude (A) greater than −100 pA and a relaxation time constant between 200 ms and 2 s were designated as dopaminergic neurones in this study (Fig.1*D*).

**Figure 1:**
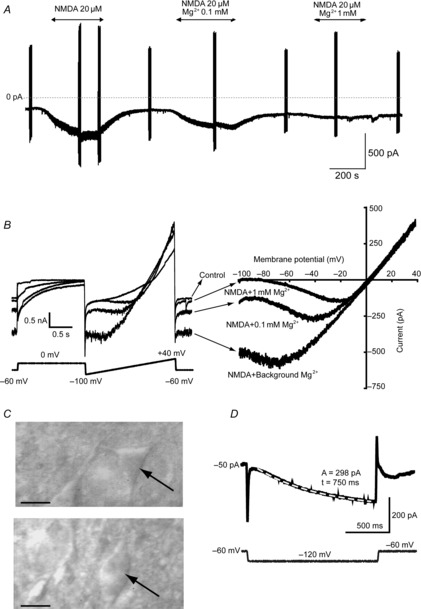
Experimental protocol and dopaminergic neurone identification *A*, example whole cell voltage clamp traces recorded from postnatal day 7 dopaminergic neurone in substantia nigra pars compacta. The neurone was voltage clamped at −60 mV and inward NMDA-mediated currents were activated by bath application of 20 μm NMDA and 10 μm glycine. Three concentrations of Mg^2+^ (0 mm, 0.1 mm and 1 mm) were tested. Bars above the current trace show the time of application of indicated solutions. Vertical lines on the NMDA-mediated current trace are current responses evoked by voltage ramps from −100 mV and +40 mV. *B*, left, expanded voltage ramp current trace from a recording illustrated in (*A*). Each current trace was obtained by averaging four repeated trails. *B*, right, example *I*_NMDA_–*V* relations obtained from (*A*), by subtracting the control current trace from currents recorded in the presence of NMDA with resultant *I*_NMDA_ values plotted *versus* membrane potential. *C*, example Differential Interference Contrast images of typical postnatal day 7 rat dopaminergic neurones. The scale represents 20 μm. *D*, example characteristic *I*_h_ current used for identification of dopaminergic neurones. White dashed line on the current trace shows single exponential fitting and fitting results are shown where (*A*) is predicted maximum *I*_h_ current and τ is the activation time constant.

### Electrophysiology and data analysis

For recordings, slices were placed in a recording chamber on the stage of an upright differential interference contrast microscope (Zeiss Axioskop, FS Germany) and continuously bathed in external recording solution (as described above but without MgCl_2_).

#### Steady-state recordings of NMDARs single channel activity and concentration jump experiments

Patch pipettes were made from thick-walled borosilicate glass (GC150F; Harvard Apparatus, Edenbridge, Kent, UK), fire polished to a final resistance of 10–15 MΩ, coated with silicone resin (Sylgard 184; USA) and filled with pipette solution containing (mm): CsCl 140; MgCl_2_ 0.5; EGTA 10; Hepes 10; ATP 1; GTP 0.5 adjusted to pH 7.4 with CsOH. Outside-out patches were made from dopaminergic neurones in SNc and voltage clamped at −60 mV. For concentration jump experiments, the rapid switching of two solutions was achieved by moving a theta-glass applicator (Jonas, 1995) driven by a piezo translator (Burleigh Instruments, Fishers Victor, NY, USA). The two channels of the theta glass contained different external solutions; 20 μm glycine and 20 μm DNQX were added to the ‘control’ solution while additional 1 mm glutamate was added to the test solution. Glutamate was applied for 1 ms or 4 s at 1 min intervals. Open tip experiments were carried out before the concentration jump experiments to map the optimal position of the applicator relative to the patch pipette (Fig.5*Ba*). The activation time course of open tip experiments was estimated with a single exponential equation and mean value of the 10–90% rise time of 10 individual experiments was 0.68 ± 0.23 ms (mean ± s.e.m.). Single channel currents were recorded using an Axopatch 200A amplifier (Axon Instruments, Foster City, CA, USA), on-line filtered at 2 kHz with an eight-pole Bessel filter, and digitized at 20 kHz using either WinEDR or WinWCP programs (Strathclyde Electrophysiology Software, George Street, Glasgow, UK). Deactivation and desensitization decay time constants were obtained by fitting current traces with a double exponential function: *I*(*t*) = *A*_1_ exp(−*t*/τ_1_) + *A*_2_ exp(−*t*/τ_2_) + *S*_s_, where τ_1_ and τ_2_ are the time constants of the fast and slow components of the decay. *I*(*t*) is the current amplitude at any given time (*t*), *A*_1_ and *A*_2_ are the current amplitude for the fast and slow exponential components and *S*_s_ refers to the steady-state current (the *S*_s_ was set to be 0 when assessing the deactivation time constant following removal of glutamate from the patch) (Fig.5*Bb* and *Cb*).

#### Whole cell voltage clamp recordings

Patch pipettes were fire polished to a final resistance of 5–6 MΩ and filled with pipette solution. Ten micromolar bicuculline methiodide, 10 μm strychnine hydrochloride, 100 nm tetrodotoxin (TTX; Ascent Scientific, Avonmouth, UK), 0.5 μm conotoxin MVIIC and 2 μm nimodipine (Sigma-Aldrich, Dorset, UK) were added to the external solution to block glycine receptors and voltage-gated sodium, N- and L-type calcium currents. For voltage-dependent Mg^2+^ block experiments, neurones were voltage-clamped at −60 mV and the membrane potential was initially stepped to 0 mV for 1.5 s to inactivate residual calcium currents. Subsequently, the holding potential was ramped from −100 mV to +40 mV at 70 mV s^−1^ (Fig.1*B*) and then returned to −60 mV. The protocol was executed in normal external solution and then repeated when the NMDA-activated current reached a steady state (Fig.1*A*). The current traces obtained in normal external solution were considered as ‘control’ and the control traces were subtracted from those recorded during NMDA application. The −100 to +40 mV ramp portion of the resulting currents was plotted against the membrane potential (Fig.1*B*). Series resistance was in the range 10–30 MΩ and was approximately 70–85% compensated. Recordings were discarded if the series resistance increased by more than 20% during the course of the recording.

### Analysis, modelling and statistics

Single NMDAR channel activity were analysed by time course fitting using SCAN in the manner described previously (Colquhoun & Sigworth, 1995; Jones & Gibb, 2005). Briefly, the duration and amplitude of channel openings were measured and then a consistent time resolution (100 μs) was applied to the data before forming distributions for the channel current amplitudes and open times and shut times using the program EKDIST (Colquhoun & Sigworth, 1995). Amplitude distributions were made for openings longer than two filter rise times (*T*_r_ = 332 μs) and fitted with the sum of three to four Gaussian components by the maximum likelihood method. Stability plots of channel amplitudes, mean open time, mean shut time and mean *P*_open_ were checked to ensure that data were stable during the recordings (Weiss & Magleby, 1989). For analysis of direct transitions between open channel amplitude levels, an amplitude-based separation of unitary currents was obtained by calculating critical amplitude values (*A*_crit_) producing an equal percentage of misclassified events between the Gaussian components fitted to the amplitude distribution (Colquhoun & Sigworth, 1995). Each amplitude level had a duration longer than 2.5 filter rise times (415 μs), without intervening closures longer than the shut time resolution (100 μs).

Two commonly used models of Mg^2+^ block were applied to the data: the sequential Mg^2+^ block model (Ascher & Nowak, 1988) (Scheme 1 and eqn 1) and trapping block model (Sobolevsky & Yelshansky, 2000) (Scheme 2 and eqn 2). These were used to assess the voltage dependence and estimate the equilibrium constant of the Mg^2+^ block. Modelling was performed in Excel and in GraphPad Prism (version 6.0). A sensitivity analysis of model parameter estimates, and correlations between them, was made by calculating how the sum of squares of the model fit to the data varied with changes in parameter estimate (using Matlab R2010b). Significant differences between model parameter estimates for different experimental data sets were assessed using 95% confidence intervals (Fig.4).

**Scheme 1:**
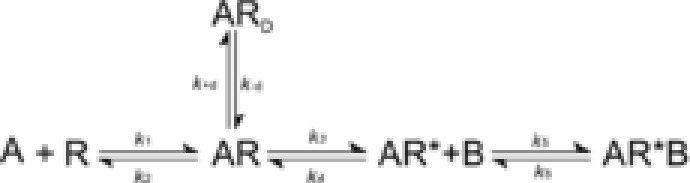
Sequential block model

**Scheme 2:**
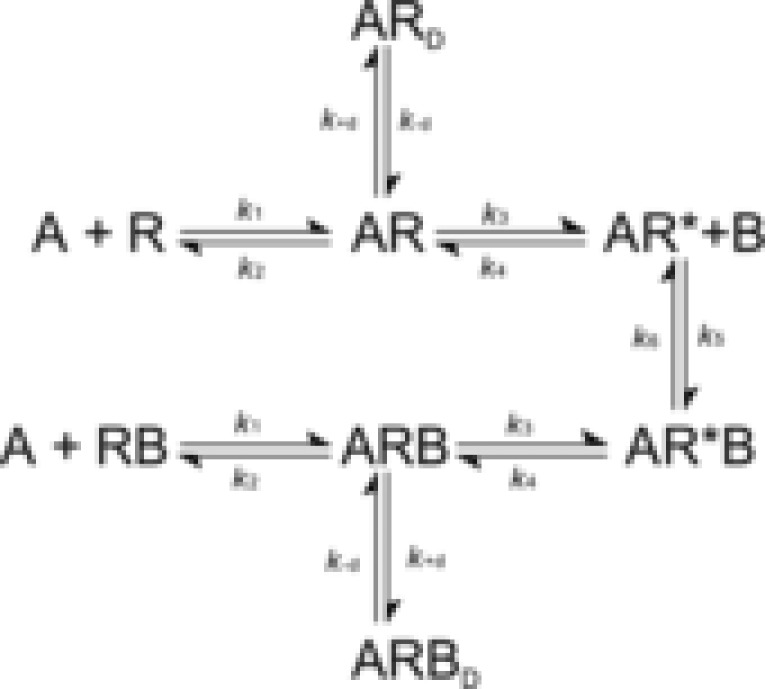
Trapping block model

Current in the presence of Mg^2+^ for the sequential block model:


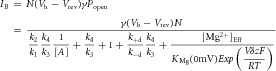
(1)

Current in the presence of Mg^2+^ for the trapping block model:


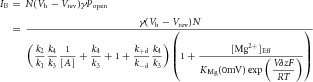
(2)

Here *k*_1_ and *k*_2_ refer to agonist binding and unbinding rates, respectively, *k*_3_ and *k*_4_ are channel opening and closing rates and *k*_5_ and *k*_6_ are Mg^2+^ microscopic association and dissociation rates. *K*_Mg_ = *k*_6_/*k*_5_ and δ gives a measure of the steepness of the voltage dependence (Ascher & Nowak, 1998). The effective [Mg^2+^]_Eff_ is the sum of the added concentration (30 μm, 100 μm, 300 μm, 1 mm or 3 mm in these experiments), plus the background Mg^2+^ in the slice. Here the voltage dependence of block was evaluated without compensation for permeant ion effects (Antonov & Johnson, 1999) so that the estimate of δ is approximately double the true electrical distance of the binding site. A is the agonist, R the receptor and ARB the blocked receptor. AR_D_ refers to the NMDAR desensitized state (Schemes 1 and 1). For simplicity, a single agonist binding reaction is used here to describe activation of the receptor and values for agonist binding rates and channel gating and desensitization were selected to give channel open probabilities and whole cell currents consistent with the observed data.

The predicted Mg^2+^ concentration–inhibition curves were obtained by fitting Mg^2+^ inhibition data with a single hyperbola of the form (in Microsoft Excel):


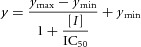


Data are reported as means ± s.e.m. Statistical comparisons were performed using either paired or unpaired Student's *t* tests. Differences were considered to be significant when *P* < 0.05 and the significant difference is indicated as * in all figures.

## Results

### Mg^2+^ block of NMDARs in postnatal day 7 substantia nigra pars compacta dopaminergic neurones

To determine the nature of the voltage-dependent Mg^2+^ block of NMDARs in P7 dopaminergic neurones, we initially performed whole cell voltage clamp recordings to measure NMDAR-mediated currents (*I*_NMDA_) in the presence of 20 μm NMDA, 10 μm glycine and six concentrations of extracellular Mg^2+^ (zero, 0.03 mm, 0.1 mm, 0.3 mm, 1 mm and/or 3 mm) (Fig.1*A*). A Cs^+^ ion-based pipette solution was used to aid in maintaining adequate voltage control. Bath application of NMDA in the absence of external Mg^2+^ induced an *I*_NMDA_ with a mean amplitude of 700.5 ± 64.6 pA (*n* = 17). Application of 0.1 mm or 1 mm external Mg^2+^ significantly reduced *I*_NMDA_ by 57.8 ± 2% (*n* = 8) and 85.9 ± 2% (*n* = 16), respectively (Fig.2*A*).

**Figure 2:**
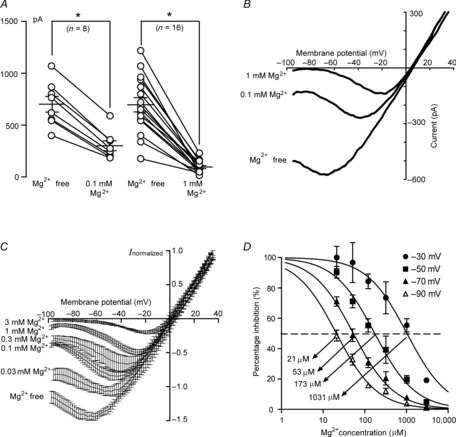
External Mg^2+^ inhibits NMDARs in dopaminergic neurones in a concentration- and voltage-dependent manner *A*, graph depicting the mean and standard error of whole cell *I*_NMDA_ measured at −60 mV in the absence and in the presence of external Mg^2+^. Open circles illustrate *I*_NMDA_ from individual experiments. *B*, example of *I*_NMDA_–*V* relations obtained from a single neurone. *C*, averaged and normalized *I*_NMDA_–*V* relations obtained from 26 neurones. *B* and *C*, Mg^2+^ concentrations indicate the added Mg^2+^ concentrations without taking background Mg^2+^ into account. *D*, concentration–inhibition curves for external Mg^2+^ inhibition of NMDARs in postnatal day 7 dopaminergic neurones. The contaminating Mg^2+^ concentration (27.9 μm) in the ‘Mg^2+^-free’ solution was estimated by fitting the Mg^2+^ blocking model, which is shown in Fig.4.

Voltage ramps from −100 mV to +40 mV were applied when *I*_NMDA_ reached a steady state to characterize the voltage dependence of the Mg^2+^ block. In the absence of external Mg^2+^, the *I*_NMDA_–*V* relation displayed a negative slope over the potential range −100 mV to −70 mV, suggesting there is a significant background concentration of Mg^2+^ in acute slices. When external Mg^2+^ concentration was increased to 0.1 mm, the NMDA responses became more voltage sensitive with a negative slope between −100 mV and −40 mV and passing maximum inward current at about −40 mV. In the presence of 1 mm external Mg^2+^, the *I*_NMDA_–*V* relation displayed a negative slope over the potential range −100 mV to −20 mV (Fig.2*B* and *C*).

To characterize the voltage dependence of Mg^2+^ inhibition of NMDA-induced currents, concentration–inhibition curves were constructed at four membrane voltages of −30 mV, −50 mV, −70 mV and −90 mV for six different Mg^2+^ concentrations (Fig.2*D*). As acute slices contain a low level of background Mg^2+^, the predicted Mg^2+^-free NMDA responses (*I*_predicted_) were estimated by extrapolating the positive part of the averaged *I*_NMDA_–*V* relations assuming a linear relationship and *I*_predicted_ was used for the calculation of percentage inhibition by each external Mg^2+^ concentration. The results suggest that external Mg^2+^ inhibits NMDARs in P7 dopaminergic neurones at −30 mV, −50 mV, −70 mV and −90 mV with the IC_50_ values, 1031 μm, 173.4 μm, 53.3 μm and 20.9 μm, respectively. These IC_50_ values infer an approximate voltage dependence corresponding to δ = 0.77. This voltage dependence is less steep than expected for GluN2A or GluN2B receptors (Kuner & Schoepfer, 1996; Wrighton *et al*. 2008) but would be consistent with a mixed population of GluN2B and GluN2D receptors (Kuner & Schoepfer, 1996; Wrighton *et al*. 2008) as suggested for SNc cells in older rats (Jones & Gibb, 2005; Brothwell *et al*. 2008).

### Triheteromeric GluN1–GluN2B–GluN2D NMDARs display a weaker voltage-dependent Mg^2+^ block than diheteromeric GluN1–GluN2B NMDARs

To investigate the voltage-dependent Mg^2+^ block properties of the triheteromeric NMDARs, we took advantage of a non-competitive subunit selective NMDAR antagonist, ifenprodil. It has an IC_50_ value of 0.34 μm for GluN1–GluN2B NMDARs, which is about 400-fold higher than IC_50_ value of 150 μm for GluN1–GluN2A, GluN1–GluN2C and GluN1–GluN2D receptors (Williams, 1993, 1995; Hatton & Paoletti, 2005). In addition, 10 μm ifenprodil inhibits about 92% of GluN1–GluN2B receptor-mediated currents, but only blocks 2–5% of NMDAR currents carried by GluN1–GluN2A, GluN1–GluN2C or GluN1–GluN2D. In addition, ifenprodil has also previously been shown to partially block both synaptic and extrasynaptic SNc dopaminergic neurone NMDA receptor currents (Jones & Gibb, 2005; Brothwell *et al*. 2008; Suarez *et al*. 2010).

First, we examined the effect of 1 μm and 10 μm ifenprodil on *I*_NMDA_ in 12 P7 dopaminergic neurones. Ifenprodil antagonizes NMDA receptors while also increasing the receptor affinity for glutamate recognition site agonists. The NMDA EC_50_ for glutamate binding sites of recombinant GluN1–GluN2B NMDARs is 30 μm and ifenprodil increases the NMDA affinity to NMDARs six times (Kew *et al*. 1996; Traynelis *et al*. 2010). Here, we chose 250 μm NMDA to activate *I*_NMDA_ in the control and in the presence of ifenprodil to avoid the complication of changes in agonist affinity. To make fair comparison, we initially confirmed that the extent of the Mg^2+^ block to NMDA-mediated currents activated by 20 μm and 250 μm are identical, suggesting that the Mg^2+^ block of NMDARs is independent of NMDA concentrations (Fig.6) (Kew *et al*. 1996). Owing to the slow kinetics of ifenprodil, two NMDA responses were obtained from each concentration of ifenprodil to check for stable inhibition and the average of the two responses was used for analysis (Fig.3*A*). Results showed that 59.2 ± 3.1% (*n* = 8) of *I*_NMDA_ was blocked by 1 μm ifenprodil while 10 μm ifenprodil caused 75.7 ± 1.9% (*n* = 12) inhibition (Fig.3*B*).

**Figure 3:**
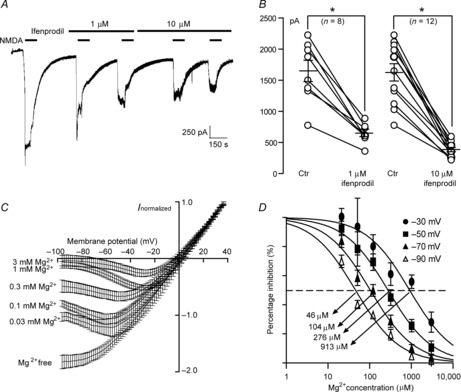
Residual whole cell NMDA currents recorded in the presence of 10 μm ifenprodil displayed weak voltage-dependent Mg^2+^ block *A*, example whole cell NMDAR-mediated current trace obtained from a postnatal day 7 dopaminergic neurone at a holding potential of −60 mV. *B*, mean and standard error of whole cell *I*_NMDA_ obtained in the absence (Ctr) and in the presence of ifenprodil is shown with the values (open circles) of *I*_NMDA_ obtained from individual experiments. *C*, averaged (means ± s.e.m.; *n* = 16) and normalized *I*_NMDA_–*V* relations recorded in the presence of 10 μm ifenprodil. *D*, Mg^2+^ concentration–inhibition curves for ifenprodil insensitive NMDARs. The calculated contaminating Mg^2+^ (27.9 μm) in acute slices was predicted using the Mg^2+^ blocking model shown in Fig.4.

Then, voltage-ramps from −100 mV to +40 mV during steady-state *I*_NMDA_ evoked by bath application of 250 μm NMDA, 10 μm glycine and 10 μm ifenprodil combined with six different Mg^2+^ concentrations (0 mm, 0.03 mm, 0.1 mm, 0.3 mm, 1 mm and 3 mm) were used to investigate the Mg^2+^ block in the presence of ifenprodil. Residual currents in the presence of ifenprodil show a characteristic region of negative slope (Fig.3*C*) and the Mg^2+^ block IC_50_ values at −30 mV, −50 mV, −70 mV and −90 mV of 913 μm, 276 μm, 104 μm and 45.9 μm, respectively (Fig.3*D*). Comparing the Mg^2+^ sensitivity of residual NMDAR currents obtained in the presence of ifenprodil (*I*_NMDA(ifen)_) with the total *I*_NMDA_, the *I*_NMDA_ were blocked more strongly by extracellular Mg^2+^ than *I*_NMDA(ifen)_ at negative voltages of −50 mV, −70 mV and −90 mV, but not at −30 mV (Table1 and Fig.7). We concluded that triheteromeric GluN1–GluN2B–GluN2D NMDARs have a lower Mg^2+^ sensitivity than GluN1–GluN2B.

**Table 1:** Parameter estimates from model fitting to Mg^2+^ concentration–inhibition curves

	Control		Ifenprodil	
Voltages	IC_50_	95% Confidence interval	IC_50_	95% Confidence interval
−30 mV	1031	799.8–1328	913.3	671.8–1242
−50 mV	173.4	145.8–199.8	275.7	205.4–370.2
−70 mV	53.3	42.2–67.3	103.7	76.0–141.5
−90 mV	20.9	15.9–27.5	45.9	32.4–65.0

### Quantification of the change in Mg^2+^ sensitivity of *I*_NMDA_ before and after application of ifenprodil

To quantitatively understand the change in Mg^2+^ sensitivity before and after application of ifenprodil, we fitted *I*_NMDA_–*V* curves obtained in the presence and in the absence of ifenprodil with the well-established trapping block model as described in Scheme 2 and eqn 2 (see Methods).

The characteristic of the Mg^2+^ trapping block model is that the agonist can dissociate from a receptor and causes the channel to close before the Mg^2+^ dissociates from the channel, leaving the Mg^2+^ ion trapped in the channel. In eqn 2, δ indicates the fraction of the membrane voltage sensed by Mg^2+^ at the blocking site and *K*_Mg_ (0 mV) refers to the dissociation constant in the absence of a transmembrane voltage. To illustrate how the voltage-dependent Mg^2+^ block parameters affect the current–voltage (*I*–*V*) relationship, the *I*_NMDA_–*V* relation is plotted for various *K*_Mg_ (0 mV) (Fig.8*A*), external Mg^2+^ concentration (Fig.8*B*) and δ values (Fig.8*C*), demonstrating that the position of Mg^2+^ in the electrical field across the membrane largely influences the Mg^2+^ block of the NMDA-mediated current at negative membrane potentials, but has little effect on current at positive membrane potentials.

The *I*_NMDA_–*V* relations were simulated using model parameters chosen to give a control open probability consistent with previous estimates (Benveniste & Mayer, 1991; Lester & Jahr, 1992; Sobolevsky *et al*. 1998). During curve fitting, *V*_rev_ reversal potential, *K*_Mg_ (0 mV) and δ, and were set to be varying while *k*_2_/*k*_1_, *k*_4_/*k*_3_ and *k*_+d_/*k*_–d_ were set to be 10 μm, 2 and 5, respectively. The background Mg^2+^ concentrations were set to be same for control and ifenprodil conditions. Using GraphPad Prism 6, we fitted our experimental data with the trapping block model (Fig.4*A* and *B*). With these approaches the background Mg^2+^ concentrations in slices were estimated in the range of 27.9 ± 1.5 μm. The δ in the control was estimated to be 0.77 ± 0.01, which is significantly greater (95% confidence interval) than 0.56 ± 0.01 in the ifenprodil condition. There is no significant difference for *K*_Mg_ (0 mV) for both conditions (Table2). In addition, we also fitted our data with the sequential model (see Scheme 1 and eqn 1 in Methods). As expected, fitting this model gives the same δ values as the trapping block model (Table2). However, the *K*_Mg_ (0 mV) values were estimated to be 0.40 ± 0.03 mm and 0.32 ± 0.02 mm for control and ifenprodil conditions, respectively (Fig.8*E* and *F* and Table2).

**Table 2:** Parameter estimates from model fitting to *I*–*V* data

	Trapping block model			Sequential block model		
Parameter	Mean	s.e.m.	95% Confidence interval	Mean	s.e.m.	95% Confidence interval
***Control***				***Control***		
*K*_m_ (μm)	5532	394	4760–6304	395	28	340–450
δ	0.77	0.01	0.74–0.80	0.77	0.01	0.74–0.80
***Ifenprodil***				***Ifenprodil***		
*K*_m_ (μm)	4525	342	4012–5246	323	24	280–403
δ	0.56	0.01	0.53–0.59	0.56	0.01	0.53–0.59

**Figure 4:**
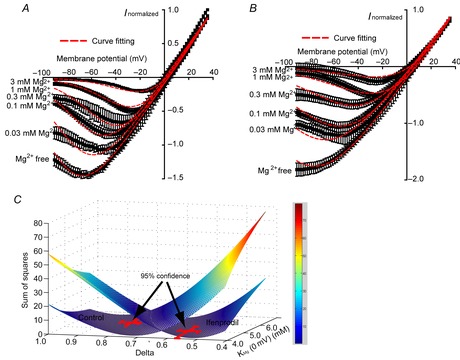
Estimation of voltage-dependent parameters of Mg^2+^ block with the trapping block model *A* and *B*, averaged and normalized *I*_NMDA_–*V* relations obtained in the absence (*A*), and in the presence (*B*), of ifenprodil are shown fitted using the trapping block model to estimate the voltage-dependent Mg^2+^ block parameters of *K*_Mg_ (0 mV) and δ and the background Mg^2+^ concentration. *C*, sensitivity analysis 3D plot illustrates the sum of squares of the trapping block model fit to the data with varied *K*_Mg_ (0 mV) and δ. The red crosses indicate the 95% confidence intervals derived from curve fitting in the control and ifenprodil conditions.

The δ value for *I*_NMDA_–*V* obtained in control conditions is significantly (95% confidence interval) greater than that obtained in the presence of ifenprodil, which is consistent with the idea that GluN1–GluN2B–GluN2D NMDARs have a weaker Mg^2+^ block than the GluN1–NR2B receptors (Table3). For both models, the sensitivity analysis of the parameter estimates indicated a negative correlation between *K*_Mg_ (0 mV) and δ as indicated by the ‘U-shaped’ profile of the sum of squares plot surface (Fig.4*C*). However, as illustrated in Fig.4*C* for the trapping block model, the minima of the sum of squares plots for the two models did not overlap between control and ifenprodil data indicating that the estimated difference in value of δ between control and ifenprodil is not model dependent.

**Table 3:** Comparison of properties between GluN1–GluN2B, GluN1–GluN2D and GluN1–GluN2B–GluN2D receptors

	GluN1–GluN2B	GluN1–GluN2D	GluN1–GluN2B–GluN2D
External Mg^2+^ block IC_50_ −70 mV (μm)	∼20	∼100	∼100
Voltage-dependent of Mg^2+^ block (δ)	∼0.9	∼0.4–0.7	∼0.56
Single channel conductance (pS)	50 main level	36 main level	50 main level
	40 sublevel	18 sublevel	40 sublevel
			20 sublevel
Deactivation time course (ms)	∼200 fast decay	∼2000	Unknown
	∼600 slow decay		
Desensitization time course (ms)	∼100 fast decay	Non-desensitizing	Unknown
	∼500 slow decay		

### The kinetics of NMDARs in postnatal day 7 rat dopaminergic neurones

As previous studies have suggested that synaptic GluN1–GluN2B–GluN2D triheteromeric NMDARs are present in P7 SNc dopaminergic neurones (Brothwell *et al*. 2008) and the composition of extrasynaptic NMDARs on these neurones is still not clear, we further investigated the possible subtypes of NR2D-containing NMDARs (GluN1–GluN2D or GluN1–GluN2B–GluN2D) present at extrasynaptic sites of these neurones, we took advantage of the unique properties of NR1-NR2D NMDA receptors, which show a very slow deactivation time course (2–5 s) and non-desensitizing properties (Wyllie *et al*. 1996; Vicini *et al*. 1998) to test the response of SNc cell NMDARs to rapid agonist application.

First, we performed steady-state single channel recordings (which represent openings between desensitized states of NMDAR activations) to confirm the subunit composition of NMDARs on P7 SNc dopaminergic neurones. All patches we recorded contained several NMDAR channels. The measured single channel amplitudes correspond to conductances (and relative areas, *n* = 5 patches from five rats) of 18.8 ± 1.0 pS (6.28 ± 3.25%), 37.8 ± 4.9 pS (25.7 ± 5.2%) and 49.0 ± 2.8 pS (72.3 ± 5.1%) (Fig. 9*B* and *C*). A small component of 30 pS openings was also seen in two of the five patches. The 49 pS and 38 pS conductance levels are characteristic of large conductance NMDA channels (composed of GluN1–GluN2A or GluN1–GluN2B) while the 38 pS and 19 pS conductance levels are characteristic of small conductance NMDA channels (composed of GluN1–GluN2C or GluN1–GluN2D) (Stern *et al*. 1992). In addition, direct transitions between conductance levels were analysed to investigate evidence for the presence of GluN2D subunit-containing receptors (Fig. 9*F*) (Wyllie *et al*. 1996). Analysis of the frequency of direct transitions between the small conductance levels showed that transitions from 38 pS to 19 pS levels occur more frequently than transitions from 19 pS to 38 pS (Fig. 9). While 55.7 ± 3.3% of direct transitions were from 38 to 19 pS, 44.3 ± 3.3% were from 19 to 38 pS (*P* < 0.05, *n* = 5). This asymmetry of direct transitions is a characteristic unique to GluN2D-containing NMDARs (Wyllie *et al*. 1996), indicating that some NMDARs in P7 SNc dopaminergic neurones contain GluN2D subunits. Our data suggest that the receptor population in P7 dopaminergic neurones is not homogeneous. Previous work shows that mRNA for GluN2A or GluN2C subunits is not detected in P7 substantia nigra (Monyer *et al*. 1994) and GluN2A and GluN2C protein is not found at this age (Dunah *et al*. 1996, 1998) suggesting that P7 dopaminergic neurones do not express GluN2A or GluN2C subunits. Taken together, out data are consistent with a previous report that suggested NMDARs on P7 SNc dopaminergic neurones express GluN1–GluN2B and GluN1–GluN2B–GluN2D NMDARs (Jones & Gibb, 2005).

Brief synaptic-like (1–4 ms) pulses of 1 mm glutamate activated macroscopic NMDAR-mediated currents from all six individual outside-out patches (Fig.5*A* and *B*). Every single patch contains several NMDA channels and they are clearly visible in all the current traces (Fig.5*A*). The single channel current traces obtained from 1 s after glutamate application (we discard the first second to avoid double openings of NMDARs) were analysed to calculate the single channel conductances of 19.8 ± 1.4 pS, 39.2 ± 6.3 pS and 50.2 ± 4.8 pS, which are consistent with conductance levels measured in steady-state single channel recordings (Fig.9) and suggested NR2B- and NR2D-containing NMDA receptors are present in every patch (Fig.5*A*). The averaged macroscopic current was obtained by averaging all traces from individual patches (Fig.5*Bb*) and fitted with a mixture of two exponential components to evaluate the NMDAR deactivation. The fast and slow component time constants were 109.5 ± 11.0 ms (*n* = 6) and 1332.5 ± 147.9 ms (*n* = 6), respectively. The relative areas of the two decay components were 62.2 ± 10.4% (*n* = 6) and 37.8 ± 6.9% (*n* = 6), respectively.

**Figure 5:**
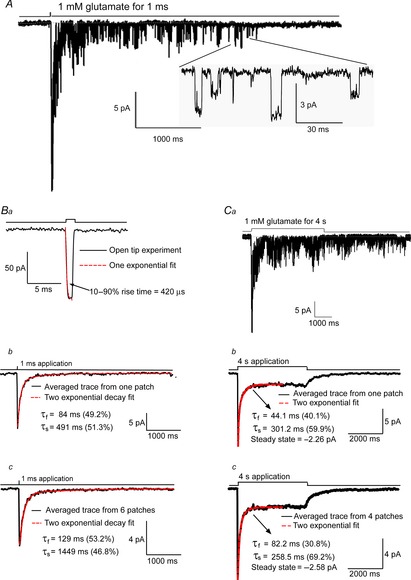
Deactivation and desensitization kinetics of NMDARs in postnatal day 7 dopaminergic neurones *A*, example concentration jump recordings obtained from an outside-out patch. NMDAR-mediated single channel current was evoked by a 1 ms pulse of 1 mm glutamate and 10 μm glycine. The expanded trace shows that both high-conductance and low-conductance NMDARs are present in this patch. *Ba*, open tip experiment used to estimate the onset of glutamate application and to optimize the best position of recording pipette. *Bb*, example macroscopic current from one individual patch. Its falling phase of the trace was well fitted with two exponential components. *Bc*, averaged macroscopic NMDAR-mediated current from six individual patches. *Ca*, example macroscopic NMDAR-mediated current obtained from an outside-out patch during a 4 s pulse of 1 mm glutamate and 10 μm glycine. The macroscopic current shows a significant desensitization following the activation of NMDARs. *Cb* and *Cc*, exemplary and averaged (*n* = 4 patches) macroscopic NMDAR-mediated currents in response to 4 s glutamate application.

During application of prolonged 4 s pulses of 1 mm glutamate to four individual patches the dopaminergic neurone NMDA receptors displayed a marked desensitization (Fig.5*Ca*). The averaged desensitization current traces from individual patches shown in Fig.5*Cb* were well fitted with a mixture of two exponential components; a fast component with time constant 97.6 ± 16.2 ms (26.8 ± 5.3% charge, *n* = 4) and slow component with time constant of 570.6 ± 71.3 ms (73.2 ± 7.9% charge, *n* = 4). The macroscopic NMDA current approached a steady-state level at 18.2 ± 7.2% (*n* = 4) of the peak current value. In addition, the deactivation kinetics after 4 ms application of glutamate were estimated to be 76.4 ± 17.9 ms (*n* = 4) for the fast component and 1563.2 ± 151.4 ms (*n* = 4) for the slow component.

In summary, all the deactivation traces are well-fitted with two exponential components and the fast components (109.5 ms for 1 ms application and 76.4 ms for 4 s application) are slower than the fast component of GluN1–GluN2A NMDARs (which are in the range 32–65 ms) but are very similar to GluN1–GluN2B NMDARs (90–250 ms) (Vicini *et al*. 1998; Wyllie *et al*. 1998), consistent with the presence of GluN2B-containing NMDARs. However, the slow decay components (1332.5 ms for 1 ms application and 1563.2 ms for 4 s application) from dopamine neurones (Fig.5) are slower than that for most recombinant GluN1a-GluN2B NMDA receptors (about 570 ms), but faster than GluN1–GluN2D NMDARs (range: 1700–5162 ms) (Table3) (Monyer *et al*. 1994; Wyllie *et al*. 1998; Vance *et al*. 2012), suggesting the possibility that in a triheteromeric GluN1–GluN2B–GluN2D receptor, the GluN2D subunit slows the GluN2B-containing NMDARs deactivation time course.

## Discussion

In the present study, we investigated the Mg^2+^ sensitivity of triheteromeric GluN1–GluN2B–GluN2D NMDARs expressed in P7 SNc dopaminergic neurones. The NMDAR-mediated whole cell currents display voltage-dependent Mg^2+^ block (Fig.2) and the Mg^2+^ sensitivity changes after blocking the ifenprodil-sensitive component of NMDAR-mediated currents (Fig.3). This is probably due to the presence of ifenprodil-resistant and low-Mg^2+^ sensitivity GluN2D-containing NMDARs as evidenced by the observed asymmetrical frequency of direct transitions between single channel conductance levels in outside-out patches (Fig.9). Computational modelling quantitatively described the Mg^2+^ sensitivity difference between the ‘normal whole cell current’ and ‘ifenprodil-resistant current’ as due to reduced voltage dependence of the Mg^2+^ block of the ifenprodil-resistant current (Fig.4). In addition, we used concentration jump experiments to study the kinetics of deactivation and the NMDAR desensitization kinetics (Fig.5). Dopamine neurone NMDAR kinetics are significantly faster than expected for GluN1–GluN2D diheteromers (Monyer *et al*. 1994; Vicini *et al*. 1998; Wyllie *et al*. 1998) but are similar to the kinetics of GluN1–GluN2B diheteromers (Vicini *et al*. 1998; Rumbaugh *et al*. 2000; Banke & Traynelis, 2003). NMDAR channel kinetics are also influenced by the presence or absence of the exon-5 N-terminal splice insert (Vicini *et al*. 1998; Rumbaugh *et al*. 2000). In dopaminergic neurones the predominant GluN1 splice variant is probably GluN1–2a or GluN1–4a (Standaert *et al*. 1994; Albers *et al*. 1999); both lack exon 5 and so have similar kinetics (Vicini *et al*. 1998; Rumbaugh *et al*. 2000). Together, the receptor pharmacology and voltage dependence of Mg^2+^ block suggest that the GluN1–GluN2B–GluN2D receptors display a weaker voltage-dependent Mg^2+^ block than GluN1–GluN2B receptors.

**Figure 6:**
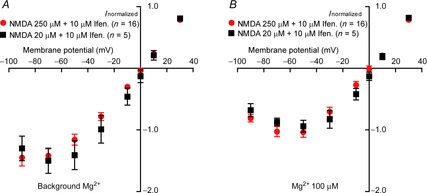
Voltage-dependent Mg^2+^ block of NMDARs is independent of agonist concentration *A* and *B*, showing the average and standard error values of normalized NMDA-mediated currents obtained at different voltages in the presence of contaminating background (*A*) or 100 μm of external Mg^2+^ (*B*) (for clarity, 300 μm and 1000 μm Mg^2+^ data are not shown). There is no significant difference in extent of Mg^2+^ block between NMDA-mediated current activated by 250 μm NMDA and 20 μm at four external Mg^2+^ concentrations (background, 100 μm, 300 μm and 1 mm of Mg^2+^), suggesting that the voltage-dependent Mg^2+^ block properties are independent of NMDA (agonist) concentration. Ifen., ifenprodil.

**Figure 7:**
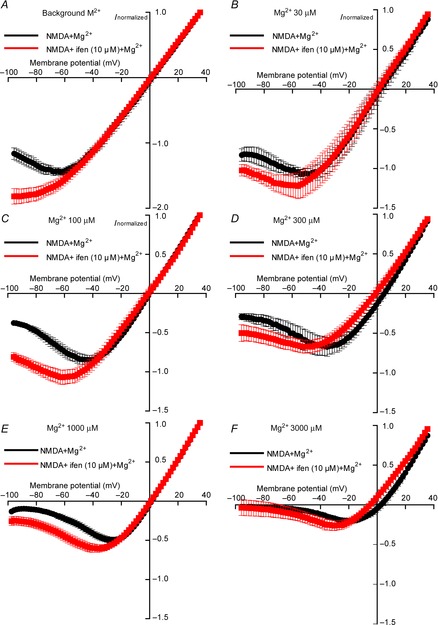
Comparison of Mg^2+^ sensitivity between residual NMDAR-mediated currents obtained in the presence of ifenprodil and normal NMDAR-mediated whole cell currents *A*–*F*, normalized and averaged *I*_NMDA_–*V* curves obtained with six different external Mg^2+^ concentration, showing a significant difference in voltage dependence of Mg^2+^ block properties between *I*_ifen-NMDA_ and *I*_NMDA_. Ifen, ifenprodil.

**Figure 8:**
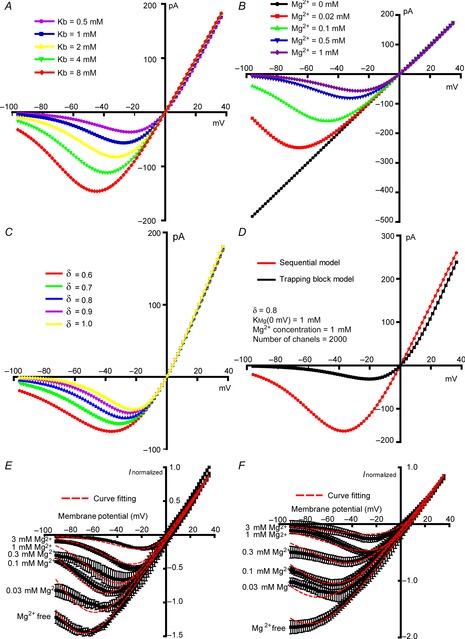
The effect of voltage-dependent parameters on predicted *I*_NMDA_–*V* relations for the sequential and trapping block models *A*–*C*, predicted *I*_NMDA_–*V* relations with the sequential block model showing *K*_Mg_ (0 mV), Mg^2+^ concentration and δ effects on the voltage-dependent block. *A*, δ, number of channels, single channel conductance, Mg^2+^ concentration and NMDA concentration were set at 0.8, 1500, 50, 1 mm and 20 μm respectively whereas in (*B*) and (*C*) δ and *K*_Mg_ (0 mV) were set at 0.8 and 1 mm respectively with the other parameters as in (*A*). *D*, simulated *I*_NMDA_–*V* relations derived from the sequential model and the trapping block models. The δ and *K*_Mg_ (0 mV) were fixed to be 0.8 and 1 mm for both models, showing that under the same conditions, the trapping block model gives stronger voltage-dependent Mg^2+^ block than the sequential block model. *E* and *F*, averaged and normalized *I*_NMDA_–*V* relations obtained in the absence (*E*) and in the presence (*F*) of ifenprodil were fitted using the sequential model to estimate the voltage-dependent Mg^2+^ block parameters of *K*_Mg_ (0 mV), δ and background Mg^2+^ concentration.

**Figure 9:**
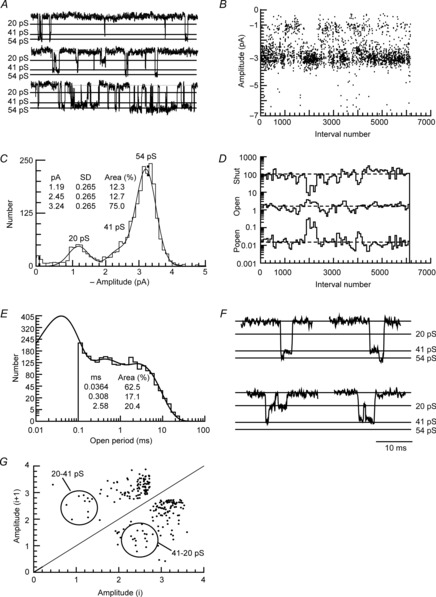
Extrasynaptic GluN2B- and GluN2D-containing NMDARs are present in postnatal day 7 substantia nigra pars compacta dopaminergic neurones *A*, examples of NMDAR single channel recordings from somatic outside-out membrane patches evoked by 10 nm glutamate and 10 μm glycine. *B*, stability plot of channel amplitudes throughout the duration of a recording. In this recording occasional ‘double’ openings are observed indicating that more than one active channel is present in the membrane patch. *C*, amplitude distribution for the patch illustrated in (*A*), fitted with the sum of three Gaussian components. The mean amplitude and relative area of each component are shown on the histogram, and correspond in this example to conductances of 20 pS, 41 pS and 54 pS. *D*, stability plots of *P*_open_, mean open time and mean shut time for the patch shown in (*A*). *E*, open time distribution for the patch illustrated in (*A*), fitted with a mixture of three exponential components. *F*, example of four types of direct transition. *G*, plot of channel amplitude before and after direct transitions (from the same patch shown in *A*). The density of points illustrates that direct transitions between 41 pS and 54 pS occur with equal frequency, while transitions between 20 pS and 41 pS are asymmetric, with 41 pS to 20 pS occurring more frequently than 20 pS to 41 pS.

### Voltage-dependent Mg^2+^ block characteristics of triheteromeric GluN1–GluN2B–GluN2D

In recombinant receptor expression systems, it has been shown that NMDARs composed of GluN1–GluN2A, GluN1–GluN2B show a higher voltage dependence of the Mg^2+^ block than receptors composed of GluN1–GluN2C and GluN1–GluN2D (Kuner & Schoepfer, 1996; Qian *et al*. 2005; Wrighton *et al*. 2008). However, different values for the voltage dependence of the Mg^2+^ block (δ value) have been reported. Kuner and Schopfer (1996) estimated the δ value for GluN1–GluN2A and GluN1–GluN2B NMDA receptors is about 1.05 and the δ value for GluN1–GluN2C and GluN1–GluN2D NMDARs is about 0.75 (Kuner & Schoepfer, 1996). A similar result was obtained by (Wrighton *et al*. 2008) for GluN1–GluN2A NMDARs (δ = 0.96) while a δ value higher than that of Kuner & Schopfer (1996) was reported for GluN1–GluN2D NMDA receptors (δ = 0.91). A possible explanation for this is that different ion concentrations were present inside and outside the oocyte membrane as the ion concentration on each side of the membrane influences the Mg^2+^ block (Antonov & Johnson, 1999). For example, in Kuner and Schoepfer's experiments, 0.18 mm Ca^2+^ was used in the external solution while 1.8 mm Ba^2+^ was used in the external solution in the experiments of Wrighton *et al*. (2008).

Fitting *I*_NMDA_–*V* relations using different models permitted us to quantify the voltage dependence of the Mg^2+^ block of NMDA channels in P7 SNc dopaminergic neurones. Here we fit the *I*–*V* relations with two commonly applied models of the Mg^2+^ block, the sequential and trapping block models (Ascher & Nowak, 1988; Sobolevsky & Yelshansky, 2000; Qian *et al*. 2005). Based on the *I*–*V* relations examined in this study, the two models provide equally good fits to the data. However, examination of the channel kinetics in the presence of Mg^2+^ suggests that a trapping block model more closely represents the Mg^2+^ block of NMDA receptors (Qian *et al*. 2005). As discussed by Qian *et al*. (2005), comparison of IC_50_ values with model-derived estimates of *K*_Mg_ provides a test between sequential and trapping block models because if the block by Mg^2+^ does not affect the NMDA receptor conformational changes associated with receptor activation and channel gating, the estimated *K*_Mg_ will be similar to the observed IC_50_. Here we measured IC_50_ values (at 0 mV, the IC_50_ 5.2 ± 1.7 mm for 20 μm NMDA and 6.0 ± 1.5 mm for 250 μm) and estimates of *K*_Mg_ using the trapping block model in the same submillimolar range (Tables1 and 2) supporting the conclusion that the Mg^2+^ block of dopaminergic neurone NMDA receptors is consistent with a trapping block model rather than the sequential block model. In addition, IC_50_ estimates are similar at 20 μm or 250 μm concentrations of NMDA, which also supports the trapping rather than sequential block model. According to these models, in the present data a δ value of 0.77 was found in control recordings, while in the presence of ifenprodil δ = 0.56 (Table2). For these two models, the value of δ is independent of the model (Table2). The block by ifenprodil is not voltage-dependent (Williams, 1993). Comparing the data presented above with recombinant NMDA receptors reveals that the δ value (0.77) obtained in the absence of ifenprodil is in the range of previous studies, is higher than the δ value for GluN1–GluN2C and GluN1–GluN2D recombinant NMDA receptors (0.75) (Kuner & Schoepfer, 1996) and smaller than that for GluN1–GluN2A and GluN1–GluN2B recombinant NMDA receptors, 0.96 (Wrighton *et al*. 2008) and 1.05 (Kuner & Schoepfer, 1996). This suggests, given the kinetic properties discussed above that native GluN1–GluN2B–GluN2D NMDARs on P7 dopaminergic neurones exhibit a less voltage-dependent Mg^2+^ block than diheteromeric GluN1–GluN2B receptors, perhaps because the GluN2D subunit L657 residue can disrupt the higher affinity-binding site for Mg^2+^ normally created by GluN2B subunits (Siegler-Retchless *et al*. 2012).

### GluN1–GluN2B and GluN1–GluN2B–GluN2D NMDARs are expressed in postnatal day 7 dopaminergic neurones

Ifenprodil is an allosteric NMDAR antagonist selective for the GluN2B subunit containing receptors. We therefore used ifenprodil to alter the proportion of current carried by GluN1–GluN2B receptors in these experiments, thus allowing investigation of whether this altered the Mg^2+^ block properties of the remaining NMDA current. Ten μm of ifenprodil inhibits recombinant GluN1–GluN2B NMDAR currents by 85–95% while the same concentration only causes less than 5% of inhibition of the NMDAR current carried by GluN1–GluN2A, GluN1–GluN2C or GluN1– GluN2D receptors (Williams, 1993; Mott *et al*. 1998). Triheteromeric GluN1–GluN2A–GluN2B NMDARs (Hatton & Paoletti, 2005) have a much smaller maximal degree of block (about 20–30%) by 10 μm ifenprodil although their IC_50_ is similar to GluN1–GluN2B (Williams, 1993; Mott *et al*. 1998; Hatton & Paoletti, 2005). The results of this study (Fig.3*A* and *B*) showed that 10 μm ifenprodil inhibits *I*_NMDA_ by 75.7 ± 1.9% (*n* = 12), which is significantly smaller than the block of recombinant GluN1–GluN2B NMDARs (Hatton & Paoletti, 2005) but greater than that observed with recombinant triheteromeric GluN1–GluN2A–GluN2B NMDARs (Hatton & Paoletti, 2005). However, it is not known whether ifenprodil will block GluN1–GluN2B–GluN2D triheteromers to the same extent. These data suggest that diheteromeric GluN1–GluN2B NMDARs are present in P7 dopaminergic neurones and that the low ifenprodil sensitivity NMDARs are probably GluN2D-containing NMDARs, diheteromeric GluN1–GluN2D and/or triheteromeric GluN1–GluN2B–GluN2D NMDARs.

However, in single channel recordings from the 15 outside-out patches that we examined in this study, all patches exhibited both large and small conductance openings (Figs 5 and 9). In addition, the five patches tested with 1–4 ms brief glutamate applications did not exhibit typical deactivation kinetics of GluN1–GluN2D NMDARs (decay time course about 2–5 s). Furthermore, immunohistochemical data indicate that NR2D-containing receptors in the midbrain are commonly present as triheteromeric GluN1–GluN2B–GluN2D receptors (Dunah *et al*. 1998). Taken together these observations support the idea that NMDARs in P7 SNc dopaminergic neurones are a mixture of diheteromeric GluN1–GluN2B and triheteromeric GluN1–GluN2B–GluN2D NMDARs. We cannot rule out the presence of the GluN1–GluN2D NMDARs but if there are any, they are probably expressed at low levels (Brothwell *et al*. 2008).

### Synaptic and extrasynaptic NMDARs on postnatal day 7 substantia nigra pars compacta dopaminergic neurones have similar subunit composition

Previous work suggested that synaptic NMDARs in P7 SNc dopaminergic neurones are composed of a mixture of diheteromeric GluN1–GluN2B and triheteromeric GluN1–GluN2B–GluN2D (Brothwell *et al*. 2008) similar to the mixed population of extrasynaptic receptors observed in this study. This observation is consistent with the idea that extrasynaptic receptors provide a reserve pool of receptors available for exchange with synaptic NMDARs and potentially could activate different signalling pathways involved in dopaminergic neurone cell survival or cell death signalling and synaptic plasticity (Newpher & Ehlers, 2008; Martel *et al*. 2009).

The NMDA receptor-mediated synaptic excitatory postsynaptic current (EPSC) of P7 rat dopaminergic neurones is described by the mixture of two exponential components with a fast decay component of 43 ms and a slow decay component of 229 ms at 30°C. (Brothwell *et al*. 2008). Assuming a *Q*_10_ of 3.5, at room temperature the synaptic NMDA EPSC fast and slow decay components may be estimated to be 164 ms and 428 ms, respectively, similar to the kinetics of GluN2B-containing receptors (Vicini *et al*. 1998; Rumbaugh *et al*. 2000). The synaptic NMDA fast decay component is similar to the fast decay for extrasynaptic NMDA receptors in these experiments, which is 128 ms (Fig.5). In addition, both fast components carry a similar proportion of the total synaptic charge transfer, being 56% and 53%, respectively. These observations suggest that in neonatal dopaminergic neurones, synaptic NMDA receptors may have similar subunit composition to extrasynaptic NMDA receptors. Interestingly, the slow decay component of the extrasynaptic receptor response in these experiments is slower than that of the synaptic EPSC (1484 ms and 428 ms respectively), which may mean that extrasynaptic NMDARs contain a higher proportion of slow NMDARs.

### Burst firing of substantia nigra pars compacta dopaminergic neurones

Midbrain dopaminergic neurones display two types of characteristic firing mode *in vivo*; tonic firing (3–8 Hz) and burst firing (14–30 Hz) (Grace & Bunney, 1984a,*b*1984b; Hyland *et al*. 2002). Burst firing of dopaminergic neurones controls dopamine release in multiple brain regions and plays an essential role in motivation, learning and attention (Schultz, 2007). *In vivo* and *in vitro* studies have shown that activation or inhibition of NMDARs, but not AMPA receptors, trigger or block burst firing, respectively (Johnson *et al*. 1992; Overton & Clark, 1997; Deister *et al*. 2009), suggesting that NMDAR activation is essential for initiation of dopaminergic neurones bursting. The preferential role of NMDARs over AMPA receptors in triggering dopamine neurone bursting is due to the voltage-dependent Mg^2+^ block properties of NMDARs (Deister *et al*. 2009). Deister *et al*., using the dynamic clamp and modelling technique, showed that removing external Mg^2+^ completely abolished burst firing. In addition, the burst firing rate increases with reducing external Mg^2+^ concentration. This is because the voltage dependence increases the hyperpolarization phase of the dopamine neurone oscillation and thus reduces the depolarization block of sodium channels (Deister *et al*. 2009). Here, we showed that extrasynaptic NMDARs on dopaminergic neurones exhibit the properties of a combination of diheteromeric GluN1–GluN2B and triheteromeric GluN1–GluN2B–GluN2D NMDARs. The latter display a weaker voltage dependence of the Mg^2+^ block. As extrasynaptic NMDARs may serve as a reserve pool for synaptic NMDARs, mediate spillover currents and be involved in certain types of synaptic plasticity, inserting GluN1–GluN2B–GluN2D NMDARs will reduce the external Mg^2+^ block of synaptic NMDARs, and thus facilitate neuronal burst firing. Moreover, extrasynaptic NMDARs and synaptic NMDARs may determine neuronal fate (Sattler *et al*. 2000). Because the relative density of synaptic and extrasynaptic GluN2D-containing receptors is probably different, the weak Mg^2+^ block of extrasynaptic GluN1–GluN2B–GluN2D NMDARs could allow more Ca^2+^ flow into neurones during action potential firing and could thus contribute to excitotoxicity.

In summary, our observations suggest that both high Mg^2+^ sensitivity (GluN1–GluN2B) and low Mg^2+^ sensitivity (GluN1–GluN2B–GluN2D) NMDARs are expressed in P7 dopaminergic neurones and the balance of evidence suggests these are located at both synaptic (Brothwell *et al*. 2008) and extrasynaptic sites (Jones & Gibb, 2005). In addition, we show that putative GluN1–GluN2B–GluN2D receptors have deactivation kinetics more similar to diheteromeric GluN1–GluN2B receptors while their Mg^2+^ sensitivity is more similar to that of GluN1–GluN2D receptors. These results may help to understand how NMDAR-dependent synaptic plasticity occurs in dopaminergic neurones and to constrain the parameters used in simulation studies of dopaminergic neurone physiology.

## Key points

- NMDAR receptors (NMDARs) are tetrameric cation channels permeable to calcium and blocked by Mg^2+^.
- Voltage-dependent Mg^2+^ block of NMDARs is crucial to several forms of synaptic plasticity and to the integration of synaptic activity with neuronal activity. Although diheteromeric GluN1–GluN2A or GluN1–GluN2B NMDARs display stronger voltage-dependent Mg^2+^ block than GluN1–GluN2C or GluN1–GluN2D NMDARs, the extracellular Mg^2+^ block properties for triheteromeric NMDARs are still elusive.
- Here, we show that in dopaminergic neurones the voltage dependence of Mg^2+^ block is less steep than previously observed in hippocampus or cortex, consistent with the presence of triheteromeric GluN1–GluN2B–GluN2D NMDARs.
- These results may help to understand the role of triheteromeric NMDARs in dopaminergic neurone synaptic plasticity and to inform simulations of dopaminergic neurone physiology.

## Additional Information

### Author's present address

Z. Huang: Department of Molecular and Cellular Pharmacology, State Key Laboratory of Nature and Biomimetic Drugs, Peking University School of Pharmaceutical Sciences, Beijing 100191, P.R. China.

## Abbreviations

GluN1, GluN2A, GluN2B, GluN2C, GluN2D, GluN3A and GluN3B: NMDAR subunits 1, 2A, 2B, 2C, 2D, 3A and 3B
*I*_NMDA_: NMDAR-mediated current
*I*_NMDA(ifen)_: residual NMDAR-mediated current in the presence of ifenprodil
NMDARs: *N*-methyl-d-aspartic acid receptors
P7: postnatal day 7
SNc: substantia nigra pars compacta
